# Radiation therapy quality in CCG/POG intergroup 9961: implications for craniospinal irradiation and the posterior fossa boost in future medulloblastoma trials

**DOI:** 10.3389/fonc.2012.00185

**Published:** 2012-12-11

**Authors:** Bernadine Donahue, Mary A. H. Marymont, Sandra Kessel, Matthew K. Iandoli, Thomas FitzGerald, Emiko Holmes, Mehmet Kocak, James M. Boyett, Amar Gajjar, Roger J. Packer

**Affiliations:** ^1^New York University School of MedicineNew York, NY, USA; ^2^Children’s Memorial, ChicagoIL, USA; ^3^Quality Assurance Review Center, LincolnRI, USA; ^4^Children’s Oncology Group, ArcadiaCA, USA; ^5^St. Jude Children’s Research HospitalMemphis, TN, USA; ^6^University of Tennessee Health Science CenterMemphis, TN, USA; ^7^Children’s National Medical CenterWashington, DC, USA

**Keywords:** medulloblastoma, radiation therapy, quality assurance, craniospinal, posterior fossa

## Abstract

**Purpose:** Associations of radiation therapy (RT) deviations and outcomes in medulloblastoma have not been defined well, particularly in the era of reduced-dose craniospinal irradiation and chemotherapy. The aim of this study is to evaluate the quality of RT on Children’s Cancer Group/Pediatric Oncology Group 9961 and analyze associations of RT deviations with outcome. **Materials and Methods:** Major volume deviations were assessed based on the distance from specified anatomical region to field edge. We investigated associations of RT deviations with progression-free survival (PFS), overall survival (OS), and explored associations with demographics and clinical variables. **Results:** Of the 308 patients who were evaluable for volume deviations, 101 patients (33%) did not have any. Of the remaining 207 patients, 50% had only minor deviations, 29% had only major deviations, and 21% had both minor and major deviations. Of the patients with major deviations, 73% had a single major deviation. The most common major deviation was in the cribriform plate region, followed by the posterior fossa (PF); PF deviations resulted from treating less than whole PF. There were no significant differences in PFS or OS between patients with deviations and those without. There was no evidence of associations of deviations with patient age. **Conclusions:** Approximately one-third of patients had major volume deviations. There was no evidence of a significant association between these and outcome. This lack of correlation likely reflects the current high quality of RT delivered in Children’s Oncology Group institutions, our strict definition of volume deviations, and the relatively few instances of multiple major deviations in individual patients. In is noteworthy that the types of PF volume deviations observed in this study were not adversely associated with outcome. As we move forward, quality assurance will continue to play an important role to ensure that deviations on study do not influence study outcome.

## INTRODUCTION

Craniospinal irradiation (CSI) provides the backbone for the definitive treatment of medulloblastoma (MB). Its planning and delivery is considered one of the most technically challenging of radiation treatments. It is generally accepted that quality control of CSI, with strict adherence to protocol guidelines, is an essential part of the appropriate treatment of MB. However, associations of radiation therapy (RT) deviations and outcome have not been defined clearly, particularly in the modern era of reduced-dose CSI and chemotherapy. The aim of this study was to investigate associations of the quality of RT and the outcome of children with posterior fossa (PF) MB treated prospectively on Children’s Cancer Group/Pediatric Oncology Group (CCG/POG) 9961 with reduced-dose CSI and chemotherapy. We describe the types of deviations identified, the association of RT deviations with progression-free survival (PFS) and overall survival (OS), and explore relationships between clinical variables, deviations, and outcome.

## MATERIALS AND METHODS

Children’s Cancer Group/Pediatric Oncology Group 9961 was a phase III trial designed for children with standard risk MB (defined as no evidence of disseminated disease on magnetic resonance imaging of the entire brain and spine or on cytologic examination of lumbar CSF, and less than 1.5 cm^2^ of residual tumor on postoperative neuroimaging) to determine if postradiotherapy cyclophosphamide would increase the rate of PFS as compared with a CCNU-containing regimen, and to determine the event-free survival (EFS), OS, and patterns of relapse with reduced-dose CSI. A dose of 23.4 Gy was to be delivered to the craniospinal axis with photons (electrons for the spinal field were allowed) in 1.8 Gy daily fractions with a boost to the entire PF of 32.4 Gy in 18 fractions for a total dose of 55.8 Gy in 31 fractions. Dose homogeneity of +7 and -5% relative to the prescription point was required throughout the central plane of the brain and PF fields, and along the longitudinal axis of the spinal cord; inhomogeneity correction for bone was not allowed. RT was to start within 31 days of surgery and be completed within 51 days of the start of RT. Details regarding the specifics of the chemotherapy delivery have previously been described (Packer et al., 2006).

Guidelines for the treatment volumes were outlined in the protocol. The cranial volume was to extend anteriorly to cover the entire frontal lobe and cribriform plate region, thus including the superior orbital tissue. The inferior border was to be at least 5 mm below the base of skull and matched to the superior border of the spinal field. A single posterior spinal field extending laterally to cover the recesses of the entire vertebral bodies with at least a 1-cm margin on either side (with extended source-to-skin distance if necessary) was preferred over adjacent spine fields; superiorly it extended to the caudad border of the cranial field utilizing appropriate matching technique. Inferiorly, the field was to extend 1–2 cm below the termination of the thecal sac as identified on sagittal MRI; a “spade” was to be used as needed to cover the nerve roots exiting the neural foramen. A 5-mm skin gap between the cranial and spinal fields was allowed, and this junction was to be moved at least twice during CSI. The boost volume included the entire PF with a 1-cm margin. Use of sagittal MRI to shape the superior border 1 cm above tentorium was encouraged. The anterior margin extended to the posterior clinoids (with shielding of the pituitary), and inferiorly the field extended to the C1/2 junction with the anterior border in that location being placed at the anterior edge of the vertebral bodies. The PF boost generally was delivered with lateral parallel-opposed portals; however, 3D planned boosts to the entire PF were allowed. Rapid central review, defined as review of the simulation films and portal images within 3 days of the institution of RT at Quality Assurance Review Center (QARC), was mandated for patients enrolled through POG; this was optional for patient enrolled through the CCG.

A retrospective analysis of the RT data was undertaken. All submitted simulation and portal images were reviewed and assessed for appropriateness of covered volumes utilizing guidelines adapted from, but not identical to, those published by Miralbell et al. (2006). Cranial simulation and portal films were evaluated for adequate coverage of the meninges. The cribriform plate and middle cranial fossa were scored separately. The distance between each of these regions and the inferior border (generally defined by a block) was measured. A major deviation was scored if the distance from the specified anatomical region to the block edge was 0 mm or if the block impinged upon the specified anatomical region; if the distance was 1–4 mm, a minor deviation was scored.

Coverage on the spinal axis was assessed by measuring the distance from the lateral recess to the field edge laterally and by the distance from the inferior edge of the thecal sac to the inferior edge of the spine field. PF volumes were assessed based on the placement of the anterior border at the posterior clinoid, the distance of the superior field edge from the tentorium, the distance of the posterior/inferior margin from the skull, and inferiorly from the C1/2 interspace. For both the spinal axis and the PF a major deviation was scored if the field edge or block abutted or transected the specified anatomical region; a minor deviation was scored if this distance was 1–5 mm. Dose and isodose distributions were not reviewed as part of this analysis, as initial central review of these revealed few deviations. The diagnostic films of patients who relapsed were reviewed in conjunction with the treatment portals to determine the location of the relapse with respect to the RT fields. The time interval from definitive surgery to start of RT was also analyzed for its influence on outcome.

### STATISTICS

Progression-free survival was defined as the time from on-study date to the earliest date of relapse, secondary malignancy, or death. OS was defined as the time from on-study date to the date of death. Patients who did not experience an event (death) for PFS (OS) were censored at their last follow-up date. PFS and OS distributions were estimated using the Kaplan–Meier method and compared among sub-populations of patients using a log-rank test. In estimating cumulative incidence of local relapse, competing events were considered to be distant relapse, secondary malignant neoplasms, and death; similarly, in estimating cumulative incidence of distant relapse, competing events were local relapse, secondary malignant neoplasms, and death.

## RESULTS

Four hundred twenty-one patients were enrolled on CCG/POG 9961 between 1994 and 2000. Forty-two patients were excluded from analysis for the following reasons: thirty with high-risk disease on central review, four with ineligible pathology, four with incomplete staging, one with an excessive delay in treatment, one with inadequate informed consent, and two without follow-up. Of the 379 remaining patients, 66 had poor quality or incomplete submission of neuroimaging and could not be fully evaluated on central review, and RT deviations could not be determined for five other patients; thus, 308 were evaluable for volume deviations. The vast majority of patients were simulated with 2D techniques utilizing photons. Eight patients were treated with protons; electrons were rarely used for treatment of spinal fields.

There were 146 major deviations which occurred in 104 patients. 101 patients (33%) did not have any RT deviations. Of the remaining 207 patients, 103 had only minor deviations, 60 had only major deviations, and the remaining 44 had both minor and major deviations (see **Table 1**). RT deviations were similarly distributed in both study arms. For 308 evaluable patients, one major deviation was identified in 76 (24.7%) patients, two major deviations in 18 (5.8%), and three major deviations in 7 (2.3%).

**Table 1:** Summary of deviations for the 308 evaluable patients.

Type of deviation	Number of patients (%)
None	101 (32.8)
Major only	60 (19.5)
Minor only	103 (33.4)
Both	44 (14.3)
Total	308 (100)

### TYPES OF VOLUME DEVIATION

The major deviations were categorized by anatomical site (see **Table 2**). The most common major deviation was related to the cribriform plate region. The next site to most frequently incur a deviation was the PF. Major deviations along the middle cranial fossa occurred in almost equal frequency to major deviations of the PF. Major deviations in the spinal fields were uncommon, as were other sites along the base or perimeter of the skull and the anterior upper cervical spine.

**Table 2:** Types of major deviations for the 308 evaluable patients.

Location	Number of patients (%)
Cribriform plate	79 (25.6)
Middle cranial fossa	25 (8.1)
Spine	3 (1)
Posterior fossa	27 (8.8)
Other*	12 (3.9)

* Includes anterior border of cervical spine, skull perimeter/occiput, anterior cranial fossa.

### ASSOCIATION OF DEVIATIONS WITH SURVIVAL

**FIGURE 1:**
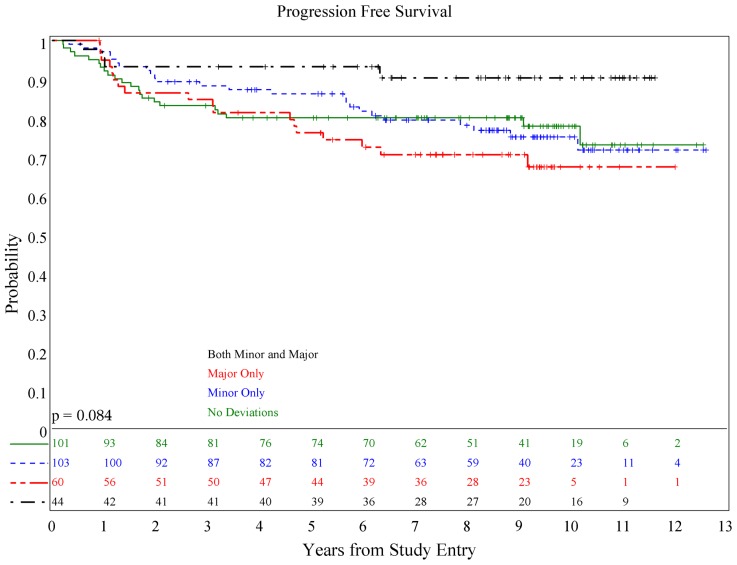
**Association of RT deviations and progression-free survival**.

**FIGURE 2:**
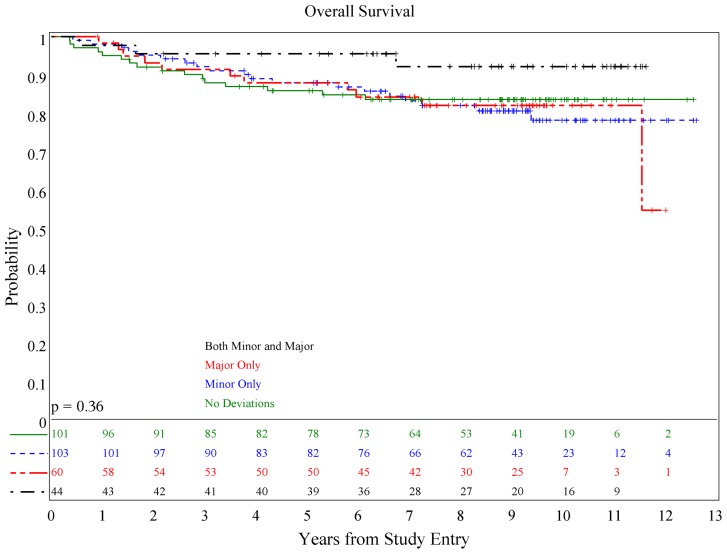
**Association of RT deviations and overall survival**.

**FIGURE 3:**
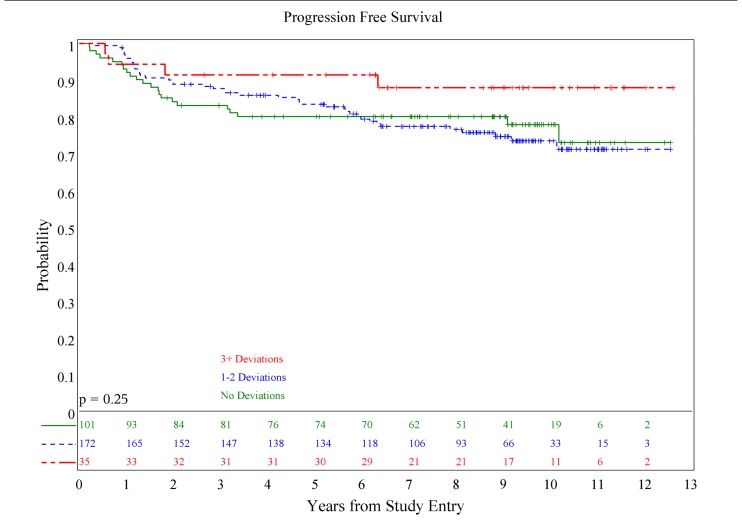
**Association of number of deviations and progression-free survival**.

**FIGURE 4:**
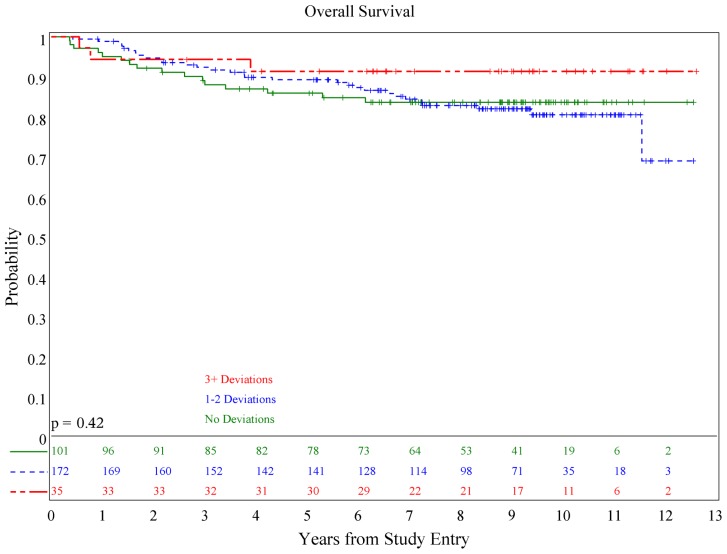
**Association of number of deviations and overall survival**.

There was no association of deviations with either PFS (*p* = 0.084) or OS (*p* = 0.36; see **Figures 1 and 2**), although patients with both minor and major deviations appear to have more favorable PFS (*p* = 0.022). Furthermore, the number of deviations (analyzed as 0, 1–2, and ≥3) did not correlate with outcome (**Figures 3 and 4**); when the number of major deviations alone was analyzed, there was also no difference in PFS or OS. Seventy-one of the 379 eligible protocol patients did not have data available to assess for RT deviations. There was no selection bias, as evidenced by no statistical difference in PFS or OS between these inevaluable patients and the 308 patients for whom this data was available.

### POSTERIOR FOSSA DEVIATIONS

**FIGURE 5:**
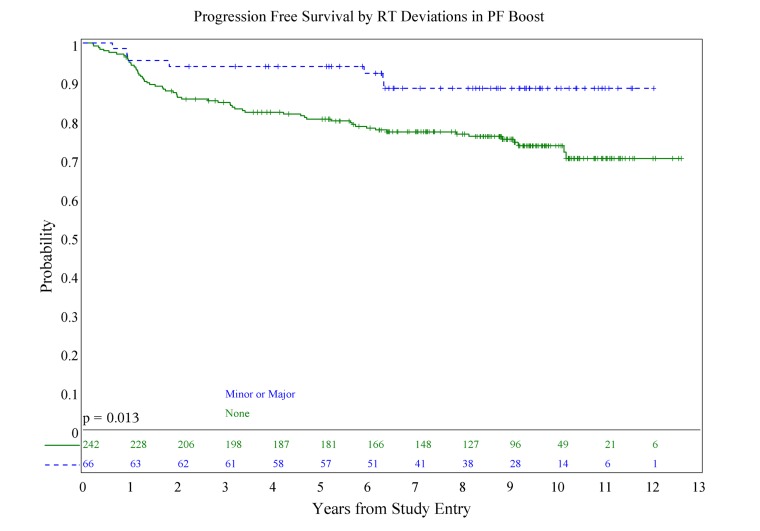
**Association of posterior fossa deviations and progression-free survival**.

**FIGURE 6:**
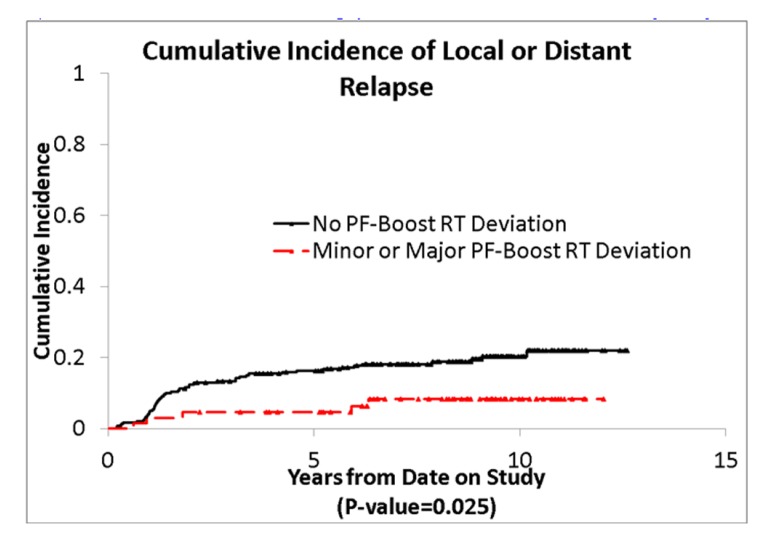
**Cumulative incidence of local and distant relapse by posterior fossa deviations**.

**FIGURE 7:**
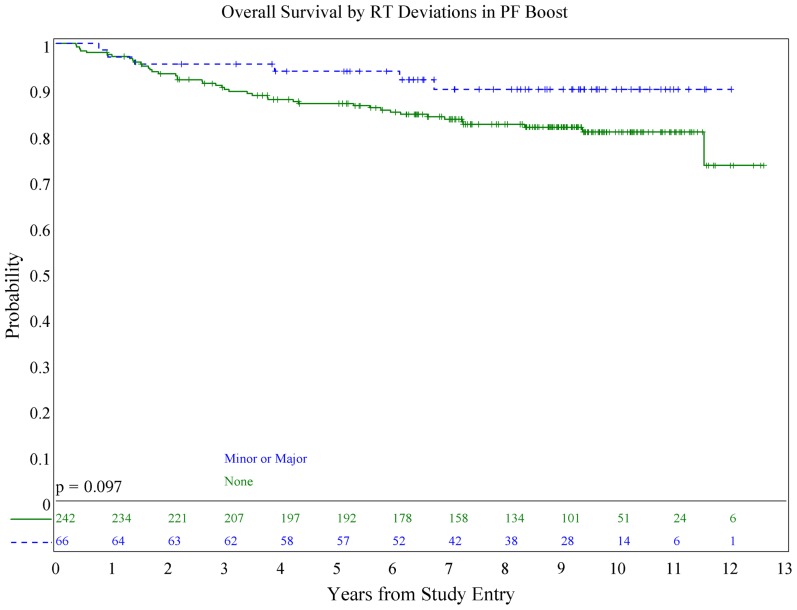
**Association of posterior fossa deviations and overall survival**.

We also evaluated whether the site of the deviation influenced outcome. There was no statistically significant association of deviations at the cribriform plate or middle cranial fossa with PFS or OS. Interestingly, however, there was a suggestion of better PFS in patients with PF deviations when major and minor deviations in this site were combined (*p* = 0.013; see **Figure 5**), and a suggestion of a lower incidence of relapse (*p* = 0.025; see **Figure 6**); there was no association with OS (**Figure 7**).

The most common PF deviation resulted primarily from treating less than the whole PF. In approximately half of these, the volume did not extend to the posterior clinoid. In the remaining cases, the field borders did not extend superiorly or posteriorly enough to allow for adequate coverage of the entire PF. In no case was the tumor bed or gross tumor shielded.

### ASSOCIATION OF CLINICAL VARIABLES AND DEVIATIONS

We evaluated the association of age on the presence and types of deviations. There was no statistically significant association of age with the number of either major or minor deviations. Additionally, we evaluated whether age was associated with RT deviations of the cribriform plate. There was no clear correlation between age and the incidence of deviations on the cribriform plate.

We investigated the association of the time elapsed from surgery to the initiation of RT with the occurrence of RT deviations. The protocol specified interval from surgery to the start of RT was 31 days. Of the 308 eligible patients, 284 (92.2%) patients started within the protocol specified interval. The median time between definitive surgery and the start of RT was 27 days (range 4–46 days), and only five patients started within a 14-day interval. The interval from surgery to the start of RT was not different between patients with RT deviation vs. patients without (Wilcoxon–Mann–Whitney test *p*-values were 0.18, 0.091, and 0.051, respectively for major, minor, and combined major and minor RT deviations).

## DISCUSSION

Medulloblastoma disseminates along craniospinal fluid pathways, and adequate coverage of the cranial and spinal contents is considered mandatory for the successful eradication of disease. To our knowledge, this is the largest review to date of the technical factors related to RT for the treatment of PF MB. The association between radiation treatment technique and patterns of failures and/or relapse rates has been explored by several authors over the past 30 years. Miralbell et al. (1997) reported a review of the whole brain RT portals in 77 patients, and in nearly half the patients, the inferior portions of the frontal and temporal lobes were judged to be “missed.” Of the 12 patients who failed in the supratentorial region, nine belonged to the group in whom the inferior portion of the brain had been underdosed. Correlation between “correct placement” of the brain field and supratentorial failure-free survival was observed.

Carrie et al. (1999) reported the impact of targeting deviations on outcome in the French Society of Pediatric Oncology (SFOP) experience with both standard risk and high-risk MB. The most frequent major deviation identified in the 169 patients evaluable for correlation between targeting deviation and the site of relapse was the cribriform plate and the lower part of the temporal lobe. Seventy percent of patients had at least one minor or major deviation, and 31% of patients had at least one major deviation. The number of major deviations in RT was correlated with the risk of tumor relapse: 67% 3-year relapse rate in patients with two deviations and 78% in patients with three major deviations. The incidence of major deviations reported in this study is similar to ours, however, comparison of the findings from the SFOP experience with our data is confounded by inclusion of high-risk cohorts, the use of neoadjuvant chemotherapy, and the start of RT at day 90 in SFOP. It is possible that in the setting of delayed RT, “marginal” misses have more of impact on outcome than when RT is delivered immediately following surgery.

In a quality assessment review of 167 patients with high-risk MB treated on POG 9031, 26% had major deviations in the brain fields, 7% in the spine, and 40% in the PF (Miralbell et al., 2006). Forty percent of patients had “inaccuracies” equivalent to our “minor deviations” in the region of the frontal lobe, and 20% in the middle cranial fossa. Including dose, more than half the evaluable patients had a major deviation, and these were not associated with a worse survival. However, of interest, patients with zero to one major deviation as compared to those with two to four major deviations, had a 5-year EFS of 71.0 vs. 61.8% (*p* = 0.30), and 5-year OS was 76.6 vs. 69.9% (*p* = 0.22), respectively.

Until recently, delivery of doses on the order of 54–55.8 Gy to the entire PF has been felt to be a necessary component for the successful irradiation of MB. The POG 9031 RT quality review showed that 40% of patients had major deviations in the PF, however, there was no correlation between deviations and survival (Miralbell et al., 2006). Several reports from single institutions in which less-than-whole PF boosts have been utilized have shown 5-year PFS on the order of 80% with rare isolated PF failures outside the involved field boost (Fukunaga-Johnson et al., 1998; Merchant et al., 2008; Carrie et al., 2009; Polkinghorn et al., 2011). The type of PF volume deviations which occurred in nearly 10% of patients on our study did not correlate with outcome. Our findings lend credence to the idea that treatment of the tumor bed and a margin to full dose, rather than the entire PF, may be sufficient, and add to the justification to study involved field vs. a PF field in the setting of combined modality therapy for standard risk MB.

It is important to emphasize that our findings should not be interpreted as lessening the value of precisely placed fields. Our study is limited by the 2D nature of the data, and the lack of correlation of deviations and outcome likely reflects both the current high quality of RT delivered in Children’s Oncology Group (COG) institutions as well as our strict definition of volume deviations. The lack of correlation between deviations and outcome could be the result of other factors, such as type II error; however, for cribriform plate deviations, the sample size was adequate to detect a reasonable difference between the PFS distributions (79 patients with major RT deviation, 83 patients with minor RT deviations), and therefore, for cribriform plate deviations, we cannot invoke type II error as an explanation for the lack of correlation. However, for deviations associated with the middle cranial fossa, the sample size was limited (25 patients with major RT deviations, 34 patients with minor RT deviations) and we may not have sufficient power to detect an existing difference. The direction of a possible difference in PFS for these deviations (middle cranial fossa) is similar to patients with PF deviations, i.e., patients with minor or major deviations seem to have better PFS and OS overall, but the difference did not reach statistical significance.

The SFOP and POG experiences suggest that radiation treatment execution does impact patient outcome. Treatment set up for this disease is challenging as a 5-mm shift in patient alignment can bring the radiation field edge into the intended target of both the central nervous system and the spine. Carrie et al. (1999) and Miralbell et al. (2006) have demonstrated that often multiple deviations must be present in the same patient in order to influence outcome. Deviations on our study were assigned at several anatomical locations, perhaps making it more difficult to demonstrate that individual deviations directly affect outcome. Furthermore, nearly three-quarters of the patients on our study who had major deviations had only one major deviation, thus potentially limiting our ability to detect an effect.

The issue moving forward is to put a strategy in place to limit or eliminate deviations on study, such that there is no question that these deviations could influence study outcome. We are entering a phase where biomarkers and targeted therapies will be incorporated into the next generation of MB studies, therefore limiting RT deviations will be important to validate new therapies moving forward. There is evidence that quality assurance in clinical trials involving RT may be influenced by RT quality even when RT is not the trial objective (Peters et al., 2010), therefore limiting RT deviations on study moving forward will be important in the next generation of clinical trials for MB.

In situations where there have been excessive deviations on study, the RT committee of the COG has moved to pre-treatment central review of treatment objects in order to limit deviations on study. With the expansion of digital transfer of both images and RT treatment objects, real time review of objects including international sites of participation is both feasible and reasonable to achieve. There are differences in defining the meningeal surface including the cribriform plate, anterior temporal lobe, and the base of the skull. These areas are also age driven in target definition, especially the cribriform region which lies at the parallax of the lens until the age of 9. Our criteria for cribriform plate deviations included not just transection of this area by a block, but abutment of the field edge to this region. Despite an increasing appreciation of the location of the cribriform plate with the advent of CT planning, this area remains at risk for being blocked, and this may reflect the bias of the treating physicians who wish to spare the lens. The frontal sinuses develop during the first and second year of life and move to their final position above the orbital ridge by 5 or 6 years of age, however, the frontal sinuses are not completely developed until late adolescence. Thus, in young children in whom the cribriform plate is anatomically closer to the lens, it becomes almost impossible to shield the lens and deliver “appropriate” dose to the cribriform.

Differences in interpretation of anatomical landmarks often contribute to study deviations. Developing age-related atlases for the meningeal surface and final phase recommendations can be very helpful and lead to successful execution of the clinical trial. Data at QARC suggests that once an atlas is developed, study deviations decrease. Coupled with real time interventional review of objects, these tools may be very helpful in limiting deviations in MB clinical trials. MB clinical trials require significant participation in both the national and international treatment community to meet accrual objectives. As we move forward with the next generation of MB studies, subsets for study in this disease will be established based on molecular phenotype, and quality assurance will continue to play an important role to ensure that deviations on study do not influence study outcome.

## Conflict of Interest Statement

The authors declare that the research was conducted in the absence of any commercial or financial relationships that could be construed as a potential conflict of interest.
